# Deficit irrigation and grafting effects on soil attributes, vegetative growth, yield components, and quality of ‘Crimson Seedless’ grapevines under a semiarid region

**DOI:** 10.3389/fpls.2026.1785340

**Published:** 2026-04-23

**Authors:** Abdel-Moety Salama, Rashid S. Al-Obeed, Abdallah Helmy, Mohamed A. Abdelsalam, Neama Abdalla, Ali R. El-Shereif, Wilfried Schwab, Mahmoud Abdel-Sattar

**Affiliations:** 1 Horticulture Department, Faculty of Agriculture, Kafrelsheikh University, Kafr El-Sheikh, Egypt; 2 Biotechnology of Natural Product, Technical University Munich, Freising, Germany; 3 Department of Plant Production, College of Food and Agriculture Sciences, King Saud University, Riyadh, Saudi Arabia; 4 Plant Biotechnology Department, Biotechnology Research Institute, National Research Centre, Giza, Egypt

**Keywords:** chemical properties, crop water productivity, irrigation, physical properties, sustainability

## Abstract

This study evaluated the combined effects of grafting and deficit irrigation (DI) on soil attributes, vegetative growth, yield components, fruit quality, and water productivity of ‘Crimson Seedless’ grapevines under semiarid conditions. It was hypothesized that the DI could enhance crop water productivity and fruit quality, especially when integrated with grafting, without a significant reduction in fruit yield. DI included five irrigation treatments: full irrigation (control), 75% of full irrigation over the season (D1), 75% of full irrigation except during the berries enlargement stage (principal growth stage 7, or BBCH 71–79), received water equivalent to the control (D2), 50% of full irrigation (D3), and 50% of full irrigation except during the berries enlargement stage, received water equivalent to control (D4), with and/or without grafting. The grapevines under deficit irrigation produced the highest crop water productivity, fruit soluble solid content (SSC), and total anthocyanins without significant impacts on fruit yield, except for D4 (severe deficit). Deficit irrigation applications saved 25%, 13%, 50%, and 26% of the water used for D1, D2, D3, and D4, respectively, compared with the control, resulting in decreased power consumption during vineyard irrigation and, subsequently, reduced irrigation costs. Moreover, soil porosity and hydraulic conductivity remained unaffected. In addition, grafted grapevines produced lower vegetative growth parameters but improved most fruit quality parameters without significantly affecting fruit yield. The present study confirmed that deficit irrigation could effectively save water and improve fruit quality without affecting fruit yield for grapevines in arid and semiarid regions. Furthermore, grafting on specific rootstock should be used as a practical approach for managing water deficit conditions.

## 1 Introduction

Grapevine (*Vitis vinifera* L.) is one of the most widely consumed fruit crops in the world, belonging to the *Vitaceae* family (Romero et al., 2020). It is the second-largest fruit crop in the world in terms of production volume and the third-largest crop in terms of planted area (75,866 km^2^), with approximately 72 million tons produced annually. Similarly, in Egypt, grapes ranked second in terms of production volume (approximately 2.0 million tons per year) and third after citrus and mango in terms of planted area (Food and Agriculture Organisation of the United Nations, 2025). Grapes are among the most important strategic crops in Egypt, as they were exported according to Food and Agriculture Organisation of the United Nations (2025), with 151,000 tonnes of grapes worth 288 million dollars in 2023, ranking fourth among the largest exported crops after citrus, fresh potatoes, and fresh onions in terms of export value. It ranked as the 11th largest exporter in 2024 among the top countries that export grapes overseas.

Water management is a crucial issue for environmental sustainability because water is a natural resource that is becoming increasingly scarce and costly due to climate change, particularly in arid and semiarid regions, especially in the Mediterranean area (Salama et al., 2021; Toumi et al., 2024). The world’s population is predicted to rise by 30% by 2050, and water shortages are also expected to worsen due to climate change, particularly in nations that heavily rely on agriculture. Therefore, irrigation strategies must be improved and developed globally. It is generally known that arid and semiarid countries, including Egypt, suffer from a shortage of water resources, which poses a major obstacle to agriculture, as the difference between water supply and demand is around 13.5 billion cubic meters of water per year (BCM/year) (Omar and Moussa, 2016; Gómez-Bellot et al., 2024). Thus, studies on improving water use efficiency have become increasingly necessary. Water-saving practices focus on reducing water consumption by increasing efficient irrigation methods, improving water productivity, improving soil health, and promoting long-term sustainability (Tan et al., 2025b). Among these practices, DI and grafting are considered promising approaches not only for water saving but also for improving yield quality, as reported by researchers on many crops, such as pistachio (Collado-González et al., 2020), grapevine (Villalobos-Soublett et al., 2022), and lemon trees (Navarro et al., 2023).

During the last two decades, deficit irrigation (DI) has been proposed as a strategy for many crops, especially in arid and semiarid regions (Salama et al., 2024). Both DI and grafting are widely used in viticulture to increase resilience to abiotic stresses (mainly drought), improve water use efficiency, and enhance grape quality. DI is considered a sustainable management practice because it not only saves the natural resources but also reduces the costs of food production and pollution, as irrigation in most new lands in Egypt depends on groundwater that is usually extracted by machines powered by diesel. The understanding of tree phenological stages and their responses to DI is essential for proper management (Gómez-Bellot et al., 2024). Crop water productivity (CWP), defined as “the ratio of the mass of marketable yield to the volume of water consumed by the crop,” was first introduced by Geerts and Raes (2009) and is regarded as a key parameter for evaluating the advantages of DI. Regulated deficit irrigation (RDI) has also been proposed for application in some deciduous fruit crops. The RDI approach involves supplying water during key growth periods while reducing irrigation during less sensitive stages (Ezzat et al., 2021). By reducing irrigation, whether in terms of timings and/or volumes, deficit irrigation not only saves water but also can increase fruit quality and reduce the cost of tree pruning and watering (Hassan et al., 2025).

The effect of DI on the vegetative growth of fruit trees, fruit yield, and fruit quality has been assessed for several horticultural crops, such as navel oranges (Nagaz et al., 2020), apricots (Ezzat et al., 2021), mango (Alhashimi et al., 2023a; Sillero-Medina et al., 2025), mandarins (Salama et al., 2024; Soto-Gómez et al., 2025), orange graves (Vanella et al., 2025), and grapevines (Martínez-Moreno et al., 2023). By the late twentieth century, investigation into the response of stone fruits to RDI began, in which trees are subjected to deficit irrigation during pit-hardening and postharvest stages, with normal watering during bud opening and fruit growth stages (Andreu-Coll et al., 2024). Thakur and Singh (2013) reported that RDI revealed no negative effects on fruit set and yield of peaches and nectarines. Deficit irrigation, alongside saving water, could also improve fruit maturity and quality (Salama et al., 2024). Pérez-Sarmiento et al. (2016) reported that advancing in fruit maturity is accompanied by an increase in fruit flavor, color, and soluble solids content (SSC) and a decrease in fruit total acidity, without a negative effect on fruit yield. Moreover, Salama et al. (2024) reported that light and moderate deficit irrigation did not show any negative effects on fruit yield, weight, and physical attributes. In addition, it improved the fruit chemical attributes, enzymatic activities, proline, and phenol contents for ‘Balady’ mandarins under the Nile Delta of Egypt. Similarly, a study on ‘Clementina de Nules’ mandarins revealed no significant reduction in fruit quality and yield due to deficit irrigation, with a 20% saving in total water consumption (Soto-Gómez et al., 2025).

Grapevines are among the fruit crops that have shown positive responses to deficit irrigation, as reported by Tan et al. (2025a), who found that RDI improved the contents of soluble solids and certain sugars such as glucose and fructose. In addition, total phenols and antioxidant activity also increased in grapevine berries compared to the control (full irrigation). Moreover, Tănase et al. (2025) also reported that moderate deficit irrigation improved fruit color intensity, total phenol content, and anthocyanin content, with no negative effects on the berry weight in three grape cultivars. Therefore, deficit irrigation applications need to be supported with additional practices or technologies that mitigate the negative effects of drought stress on fruit trees, especially during sensitive stages (El Shafei et al., 2023; Mostafa et al., 2024; Romero et al., 2025).

Under water-limited conditions, efficient irrigation management is crucial for maintaining grape productivity and quality. However, several key questions remain regarding how DI can enhance water use efficiency and productivity. To what extent can DI affect the soil attributes? To what extent can combined DI and grafting affect water productivity, vegetative growth, fruit yield, and fruit quality? Addressing these questions is particularly important for sustainable viticulture in arid and semiarid regions. Therefore, this study aimed to evaluate the effects of grafting and different levels of deficit irrigation on soil attributes, vegetative growth, yield, fruit quality, and irrigation water productivity of ‘Crimson Seedless’ grapevines under semiarid conditions.

## 2 Materials and methods

### 2.1 Experimental site and plant materials

**Figure 1:**
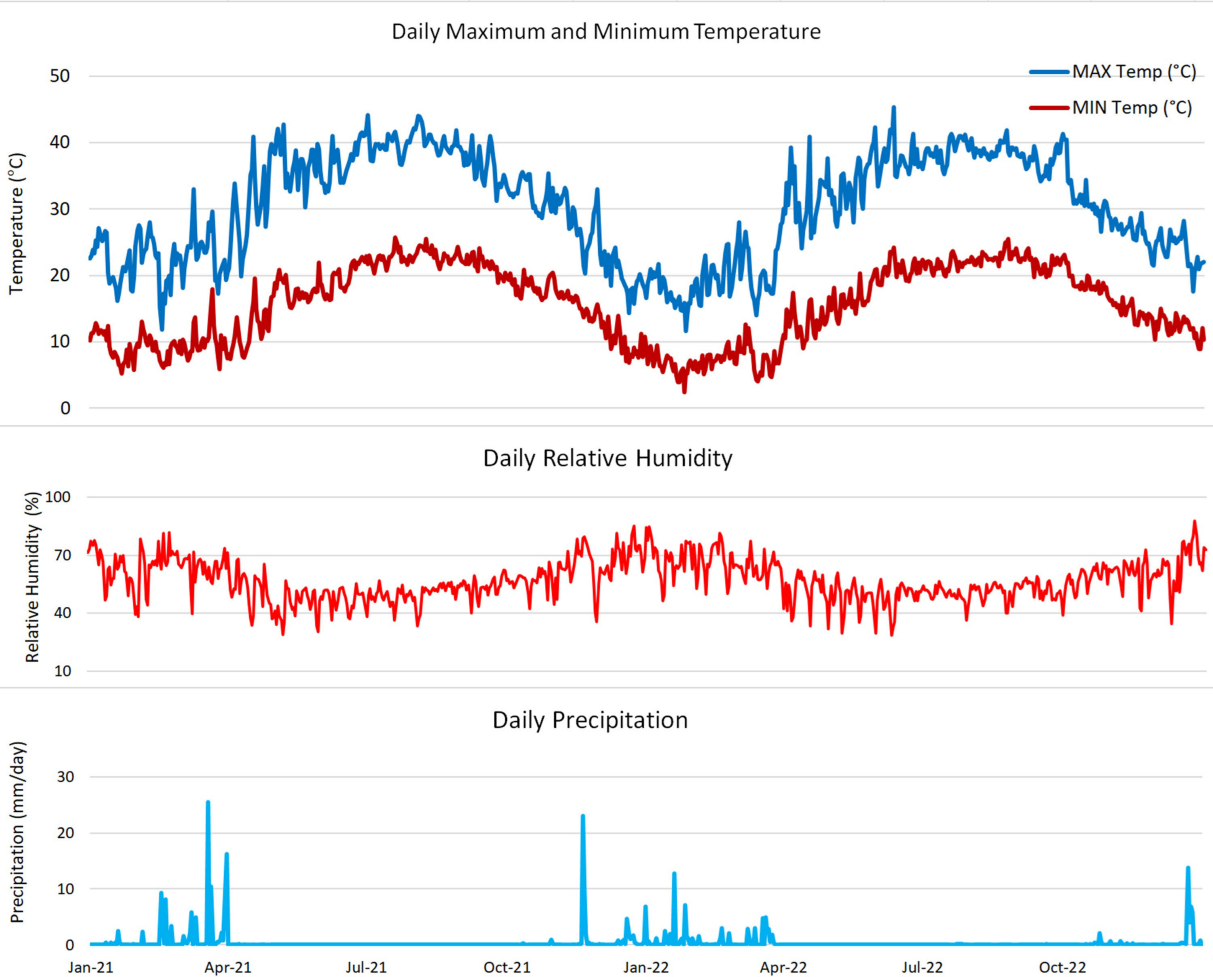
Relative humidity, maximum and minimum temperatures, and precipitation at the study site during the experimental period.

The experiment was conducted during two seasons, 2021 and 2022, to investigate the effects of grafting and deficit irrigation levels on the ‘Crimson Seedless’ grape cultivar in a private vineyard located in the Al-Khatatbeh district, Menoufia Governorate (30°22′31.4″ N, 30°38′55.3″ E), Egypt. The experimental meteorological data were recorded daily, including maximum and minimum temperatures (°C), relative humidity (%) at 2 m height, and precipitation (mm day^−1^) at the experimental site (**Figure 1**). The vines were 5 years old, cultivated at a spacing of 2 m × 3 m under a drip irrigation system. The soil was sandy, with a pH of 7.60 and a soil electrical conductivity (EC) of 2.05 dS m^−1^. Irrigation was conducted via a drip irrigation system with 8 emitters/4 L h^−1^, with four emitters on each side. In 75% treatments, two emitters were closed, leaving three emitters on each side, whereas in the 50% treatments, four emitters were closed, leaving two emitters on each side.

### 2.2 Irrigation requirement

The amount of applied irrigation requirement (IR) for grapevines during the phenological period (2021and 2022 seasons) was calculated according to Equation 1, as recommended by Keller and Karmeli (1974):

(1) $$\text{IR}=[\frac{\text{A}\times \text{CF}\times \text{ETo }\times \text{ Kc }}{10^{7}\times \text{Ea }}]+\text{LR}$$

Where IR is the irrigation water requirements (m^3^ ha^−1^ day^−1^), ETo is the reference evapotranspiration (mm day^−1^), Kc is the AO-56 crop factor of grapevine, *A* is the area irrigated (m^2^), Ea is the irrigation efficiency (%), where 90% drip irrigation, LR ids the leaching requirement for salt leaching (mm), and CF is the covering factor that reflects the percentage of soil covered by crop canopy for grapevines (35%).

ETo was calculated according to the FAO 56 Penman–Monteith equation (Allen et al., 1998). ETc was calculated as the Equation 2:

(2) ETc = Kc × ETo

Where Kc (dimensionless) is the crop coefficient, which was 0.25, 0.45, 0.60, 0.70, 0.70, 0.65, 0.55, and 0.45 for each month from March to October, respectively, according to Food and Agriculture Organisation of the United Nations (1984).

### 2.3 Experimental design and treatments

**Figure 2:**
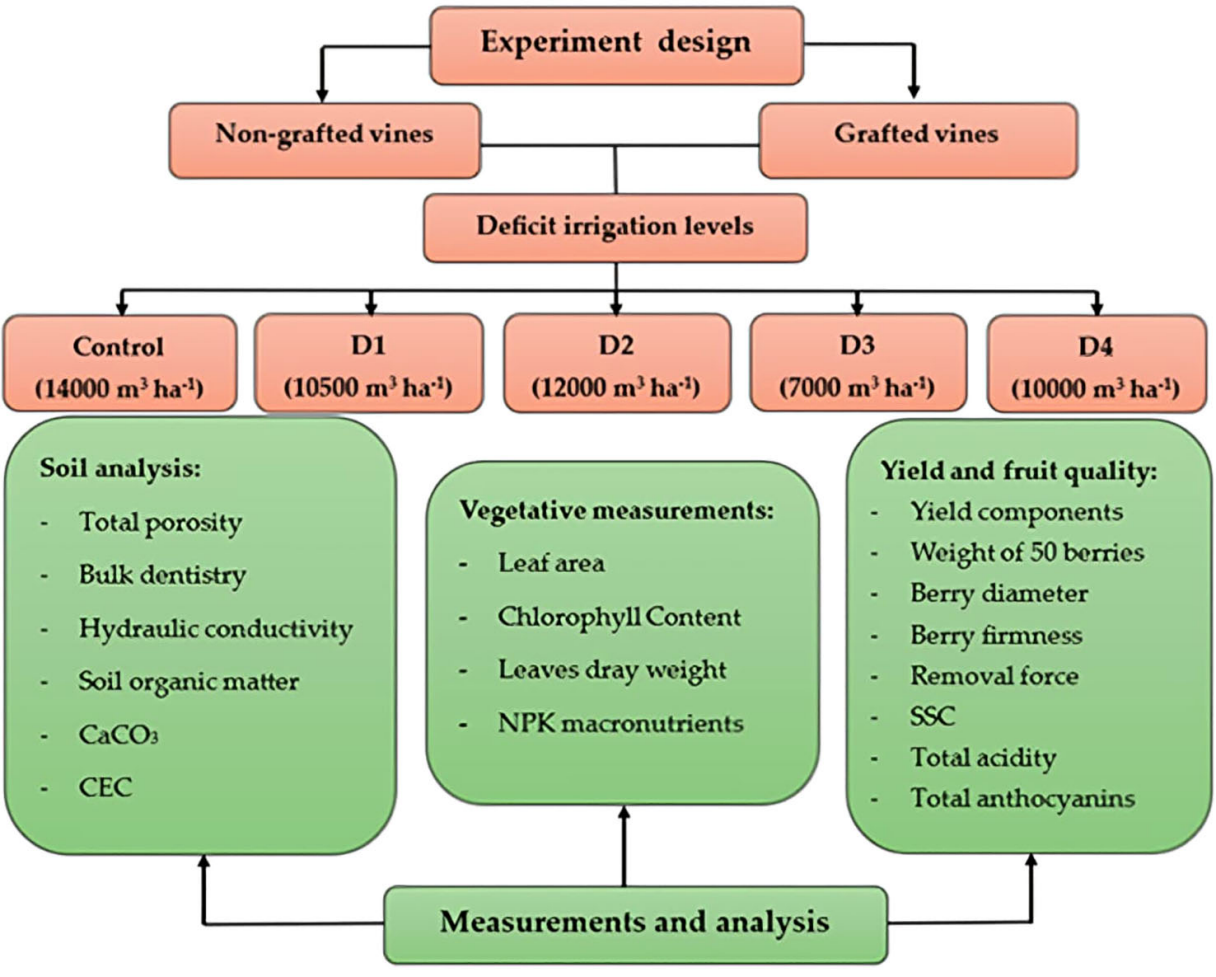
Designs and treatments for the experiment.

In this experiment, 10 treatments were designed and applied to a total of 60 vines in a split-plot design, with grafting as the main plot and irrigation treatments as subplots. Each treatment was replicated three times, with two vines per replicate, for a total of 60 vines (grafted applications [grafted and nongrafted] × five irrigation treatments × three replicates × two vines/replicate). The main plots included nongrafted vines and grafted onto 110 Richter (*Vitis rupestris × Vitis berlandieri*) rootstock. Five regulated deficit irrigation regimes were applied in the subplots: full irrigation according to regional requirements per season (14,000 m3 ha^−1^) (control), 75% of control irrigation over the season (10,500 m^3^ ha^−1^) (D1), 75% of control irrigation except during berry enlargement, when full irrigation was applied (12,000 m^3^ ha^−1^) (D2), 50% of control irrigation over the season (7,000 m^3^ ha^−1^) (D3), and 50% of control irrigation except during berry enlargement, when full irrigation was applied (10,000 m^3^ ha^−1^) (D4). Each vine in the control group was irrigated with eight emitters (4 L h^−1^), four on each side. For D1 and D2, six emitters per vine were used, three on each side. For D3 and D4, four emitters per vine were used, two on each side. In D2 and D4, full irrigation was provided during fruit enlargement, corresponding to principal growth stage 7 (BBCH 71–79) as defined by Lorenz et al. (1995). This was achieved by supplying two emitters per vine in D2 and four emitters per vine in D4, exceeding the number of emitters used during the fruit enlargement stage. Under the study conditions, vines received 275 h of irrigation per season: 104 h during fruit enlargement (from May 24 to July 25) and 171 h during the remainder of the season (**Figure 2**).

### 2.4 Measurements and determinations

#### 2.4.1 Soil sampling

Soil samples were collected before the experiment began (January) for physical and chemical analyses according to Dane and Topp (2020) and Sparks et al. (2020), respectively. Soil samples were taken (0–30 cm) at the end of the growing season (during August) from all studied irrigation treatments (D1, D2, D3, and D4, along with the control). Soil samples were collected as composite soil samples for measuring the following soil parameters: bulk density (g cm^−3^), total porosity (%), hydraulic conductivity (cm h^−1^), soil organic matter (g kg^−1^), calcium carbonate content (CaCO_3_, g kg^−1^), and cation exchange capacity (cmol_c_ kg^−1^).

#### 2.4.2 Vegetative growth and chemical measurements of leaves

From the middle of each vine at the beginning of the veraison stage, according to the methodology of Abdel-Sattar et al. (2022), 20 leaves were collected from the main shoots opposite the basal clusters to estimate vegetative growth and chemical characteristics of the leaves. Leaf area was measured using a planimeter (LI-3100 leaf area meter, LI-COR, Lincoln, Nebraska, USA) for a total of 30 leaves, representing the four dimensions of the tree (Teobaldelli et al., 2019). Chlorophyll content was determined using the same leaves as the Soil Plant Analysis Development (SPAD) unit without a chlorophyll content meter (CCM-200 plus, OPTI SCIENCES, Hudson, NY, USA). Leaves NPK macronutrients were determined by wet digestion of a 0.2-g sample of dried leaves petioles using a mixture of concentrated sulphuric acid and perchloric acid, followed by heating the mixture until a clear solution was obtained (Ryan and Asephan, 2001). The resulting solution was then quantifiably transferred into a 100-ml measuring flask and diluted with distilled water for further analysis. The percentages of N, P, and K were calculated according to Mertens (2005).

#### 2.4.3 Fruit physical properties

When berries reached full color, based on a visual color assessment, a sample of 18 clusters per treatment that were free of any discernible defects (three clusters/vineyard) was harvested in the first week of September to assess fruit physical characteristics. The weight of 50 berries (g) was measured. Berry diameter was measured using a Vernier caliper (mm, Normalcy International Ltd., Marseille, France), as the average of 30 berries per treatment. The penetrometer (push-full dynamometer, model FD101, Facchini srl, Alfonsine, Italy) was used to evaluate fruit firmness with a probe 1 mm in diameter. For each treatment, 30 berries were measured on both sides, without removing the fruit peel, and measurements were taken in the equatorial region. Berry firmness was recorded in Newtons (N cm^2^. The removal force (N) was calculated using a penetration pressure gauge on three berries (Balic et al., 2014).

#### 2.4.4 Fruit chemical properties

To determine the chemical properties, another random sample of 50 berries per vine (selected from the top, middle, and bottom of each cluster) was collected for juice extraction using a commercial juicer to obtain a homogeneous juice sample. A Bellingham and Stanley digital refractometer (Model: RFM 340-T, Washington, DC, USA) was used to estimate the SSC (%) at 20 °C. To determine the total acidity (TA, %), an automatic titration (785 DMP Titrino, Metrohm, Filderstadt, Germany) using 0.1 N NaOH up to pH 8.2 was performed, and the results were expressed as a percentage of tartaric acid, since it is a dominant acid in grapes (AOAC, 2019). The total anthocyanins were quantified using spectrophotometry at 535 nm after being extracted in acidified methanol (HCl 1.5 mol/L + 99% ethyl alcohol). A flask containing 3 g of berry skin was filled with 25 ml of the acidified methanol solution. The flask was then kept in the dark for 24 h before spectrophotometric analysis using a double-beam UV/visible spectrophotometer (Libra S80 PC, Biochrome Ltd., Cambridge, UK). The results were expressed as milligrams per gram of berry peel, as malvidin-3-glucoside (von Wettstein, 1957).

#### 2.4.5 Yield components

Yield components were estimated by determining yield, crop water productivity, irrigation cost, and income for each tree. On the harvest day, when berries reached full color, the total yield was calculated as kilograms per vine. A sample of 20 clusters per vineyard was harvested, and fruit samples were submitted to the Laboratory of Physiology and Breeding of Horticultural Crops, Faculty of Agriculture, Kafrelsheikh University, Kafrelsheikh, Egypt, for estimation of fruit quality attributes. Crop water productivity was calculated as the ratio between fruit yield and applied irrigation water according to Fernández (2020), using Equation 3:

(3) $$\text{Crop water productivity}=\frac{\text{Fruit yield }(\text{kg})/\text{tree}}{\text{Water applied }({\text{m}}^{3})/\text{tree}}$$

Irrigation costs (Irrig. Cost) were calculated based on the cost of diesel fuel used for irrigation (Egyptian pound [EGP] ha^−1^ year^−1^). The income of each tree was also estimated by multiplying the fruit yield (kg) by the price per kilogram based on the farm gate price, using Equation 4:

(4) Income (for each tree)=fruit yield (kg)*price (EGP)

### 2.5 Statistical analyses

All the collected data relating to different parameters of ‘Crimson Seedless’ grapes were subjected to two-way ANOVA using SAS version 9.13 (2008). A split-plot design within a randomized complete block design was used for the experiment. The means of the treatments were compared using Duncan’s multiple range test (LSR) when *p* < 0.05, following the methods outlined by Montgomery (2017).

## 3 Results

### 3.1 Water deficit levels and studied soil parameters

To better understand how water deficit impacts soil properties, selected soil parameters are listed in **Table 1**, including bulk density (g cm^−3^), total porosity (%), hydraulic conductivity (cm h^−1^), soil organic matter (g kg^−1^), calcium carbonate content (CaCO_3_, g kg^−1^), and cation exchange capacity (CEC; cmol_c_ kg^−1^). Data from this table can be compared with the control, which shows studied soil chemical parameters (i.e., soil organic matter [SOM], calcium carbonate content, and CEC) were significantly influenced by water deficit treatments. The most striking observation to emerge from the data comparison was that both total porosity and hydraulic conductivity did not show a significant difference among treatments, whereas a significant response was recorded for soil bulk density.

**Table 1:** Impact of different water deficit levels on soil physical and chemical attributes.

Treatments	Total porosity (%)	Hydraulic conductivity (cm h^−1^)	Bulk density (g cm^−3^)	SOM (g kg^−1^)	CaCO_3_ (g kg^−1^)	CEC (cmol_c_ kg^−1^)
Control	96.51 a	0.0011 a	1.32 a	6.4 a	28.2 a	11.59 a
D1	98.13 a	0.0011 a	1.23 b	5.4 c	28.0 b	11.14 b
D2	94.91 a	0.0026 a	1.35 a	5.9 b	28.0 b	11.14 b
D3	92.09 a	0.0010 a	1.30 a	3.9 d	27.8 c	11.06 b
D4	99.12 a	0.0010 a	1.31 a	3.9 d	27.6 d	11.14 b
*F*-test	ns	ns	^**^	^**^	^**^	^*^

SOM, soil organic matter; CEC, cation exchange capacity; control, 14,000 m^3^ ha^−1^; D1, 75% of control (10,500 m^3^ ha^−1^); D2, 75% of control except at the berries enlargement stage, similar to control (12,000 m^3^ ha^−1^); D3, 50% of control (7,000 m^3^ ha^−1^); D4, 50% of control except at the berries enlargement stage, similar to control (10,000 m^3^ ha^−1^).

Different letters indicate significant differences among treatments at test (*p* < 0.05). *Significant at 0.05 level, **Significant at 0.01 level according to F-test.

### 3.2 Chlorophyll content, leaf area, and dry weight of leaves

Data in **Table 2** show that grafting, irrigation levels, and their combination have a significant (*p* < 0.05) impact on leaf area, chlorophyll content, and leaf dry weight. In both seasons, the nongrafted vines produced the highest significant leaf area and dry weight, whereas grafted vines produced the highest chlorophyll content; however, in the second season, the difference in dry weight between grafted and nongrafted vines was not significant. Regarding irrigation treatments, there were no significant differences in chlorophyll content except for D2, which produced the lowest value in the first season, whereas in the second season, the D2 treatment produced the highest chlorophyll content compared with the control and D3 treatments. In addition, D1, D2, and D4 produced the highest leaf area compared with the control and D3 in the first season; however, there were no significant differences in the second season. Moreover, no discernible variances were found in dry weight in both seasons. Regarding the interaction, grafted vines under the control treatment had the highest chlorophyll content in the first season, whereas nongrafted vines under irrigation levels D2 and D3 had the lowest. In the second season, the combination of nongrafted vines under the control, D3, and D4 treatments produced the lowest chlorophyll content; however, there were no significant differences among the remaining combinations. Moreover, the combination of nongrafted vines with D1 had the largest leaf area in the first season, whereas grafted vines with deficit irrigation (D3 and D4) had the smallest; however, there were no discernible changes in the second season. Additionally, the combination of nongrafted vines and D1 produced the highest leaf dry weight, while grafted vines under D3 produced the lowest in the first season; however, in the second season, the only significant difference was observed in the combination of grafted vines and the control, which produced the lowest leaf dry weight.

**Table 2:** Effect of grafting, irrigation levels, and their combination on chlorophyll content (SPAD value), leaf area, and dry weight of grape leaves during both seasons.

Treatment	Chlorophyll content (SPAD unit)		Leaf area (cm^2^)		Leaves dry weight (g)	
	2021	2022	2021	2022	2021	2022
Nongrafted	9.15 b	10.01 b	1,058.46 a	785.8 a	11.4 a	10.49 a
Grafted	11.02 a	11.87 a	676 b	766 a	9.18 b	8.34 b
*F*-test	^**^	^**^	^**^	ns	^**^	^*^
Control	11.1 a	10.37 b	792.16 b	738.5 a	10.2 a	8.21 a
D1	10 ab	11.28 ab	983.16 a	825.5 a	11.36 a	10.73 a
D2	9.21 b	11.75 a	882 ab	780.66 a	9.86 a	8.68 a
D3	9.61 ab	10.85 ab	818.83 b	788.5 a	10.06 a	9.98 a
D4	10.51 ab	10.43 b	860 ab	746.33 a	9.95 a	9.46 a
*F*-test	^*^	^*^	^*^	ns	ns	ns
Nongrafted						
Control	9.56 bcd	9.48 c	959.33 bcd	734 a	10.5 bc	10.7 a
D1	10.06 bcd	10.35 bc	1,170 a	826.66 a	13.3 a	11.56 a
D2	8.2 d	11.25 ab	977 bc	722 a	10.53 bc	8.23 ab
D3	8.6 cd	9.4 c	1,084 ab	821 a	12.2 ab	11.5 a
D4	9.33 cd	9.56 c	1,102 ab	825.33 a	10.46 bc	10.46 a
Grafted						
Control	12.63 a	11.26 ab	625 ef	743 a	9.9 bcd	5.73 b
D1	9.93 bcd	12.21 a	796.33 cde	824.33 a	9.43 cd	9.9 a
D2	10.23 bcd	12.25 a	787 de	839.33 a	9.2 cd	9.13 ab
D3	10.63 abc	12.31 a	553.66 f	756 a	7.93 d	8.46 ab
D4	11.7 ab	11.3 ab	618 ef	667.33 a	9.43 cd	8.46 ab
*F*-test	^*^	^*^	^*^	ns	^*^	^*^

Control, 14,000 m^<s>3</s>^ ha^−1^; D1, 75% of control (10,500 m^3^ ha^−1^); D2, 75% of control except at the berries enlargement stage, similar to control (12,000 m^3^ ha^−1^). D3, 50% of control (7,000 m^3^ ha^−1^); D4, 50% of control except at the berries enlargement stage, similar to control (10,000 m^3^ ha^−1^).

Different letters indicate significant differences among treatments at test (*p* < 0.05). Data are presented as means of three replicates (*n* = 3). *Significant at 0.05 level, **Significant at 0.01 level according to F-test.

### 3.3 Leaf content of macronutrients

**Figure 3:**
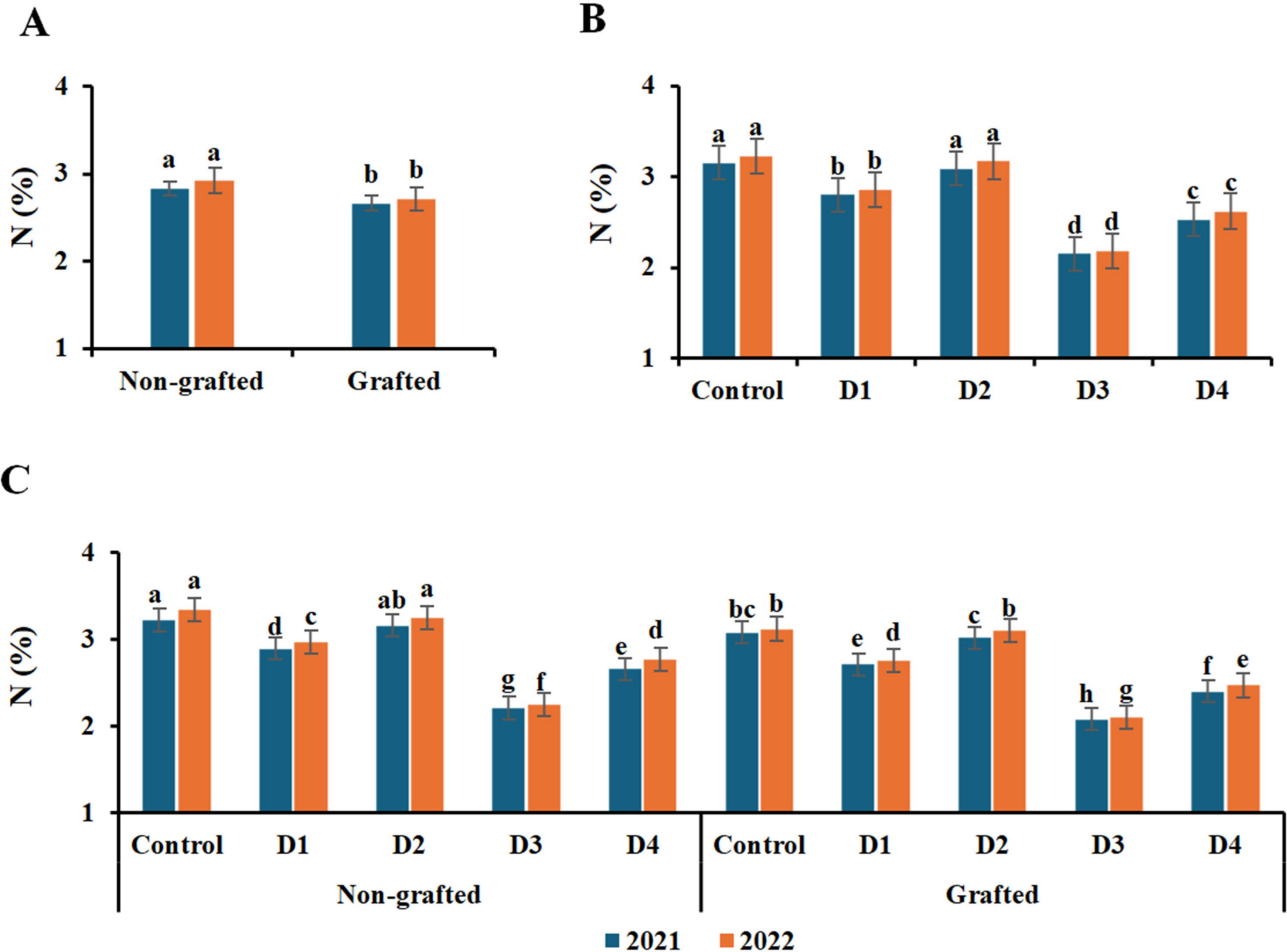
Effect of grafting **(A)**, irrigation levels **(B)**, and their combination **(C)** on N content during both seasons. Control; 14000 m^3^ ha^−1^), D1 (75% of control; 10500 m^3^ ha^−1^), D2 (75% of control except berries enlargement stage like control; 12000 m^3^), D3 (50% of control; 7000 m^3^ ha^−1^), D4 (50% of control except berries enlargement stage like control; 10000 m^3^); Different letters indicate significant differences among treatments at test (*P* < 0.05). Data are presented as means of three replicates (n = 3) ± standard error (SE).

**Figure 4:**
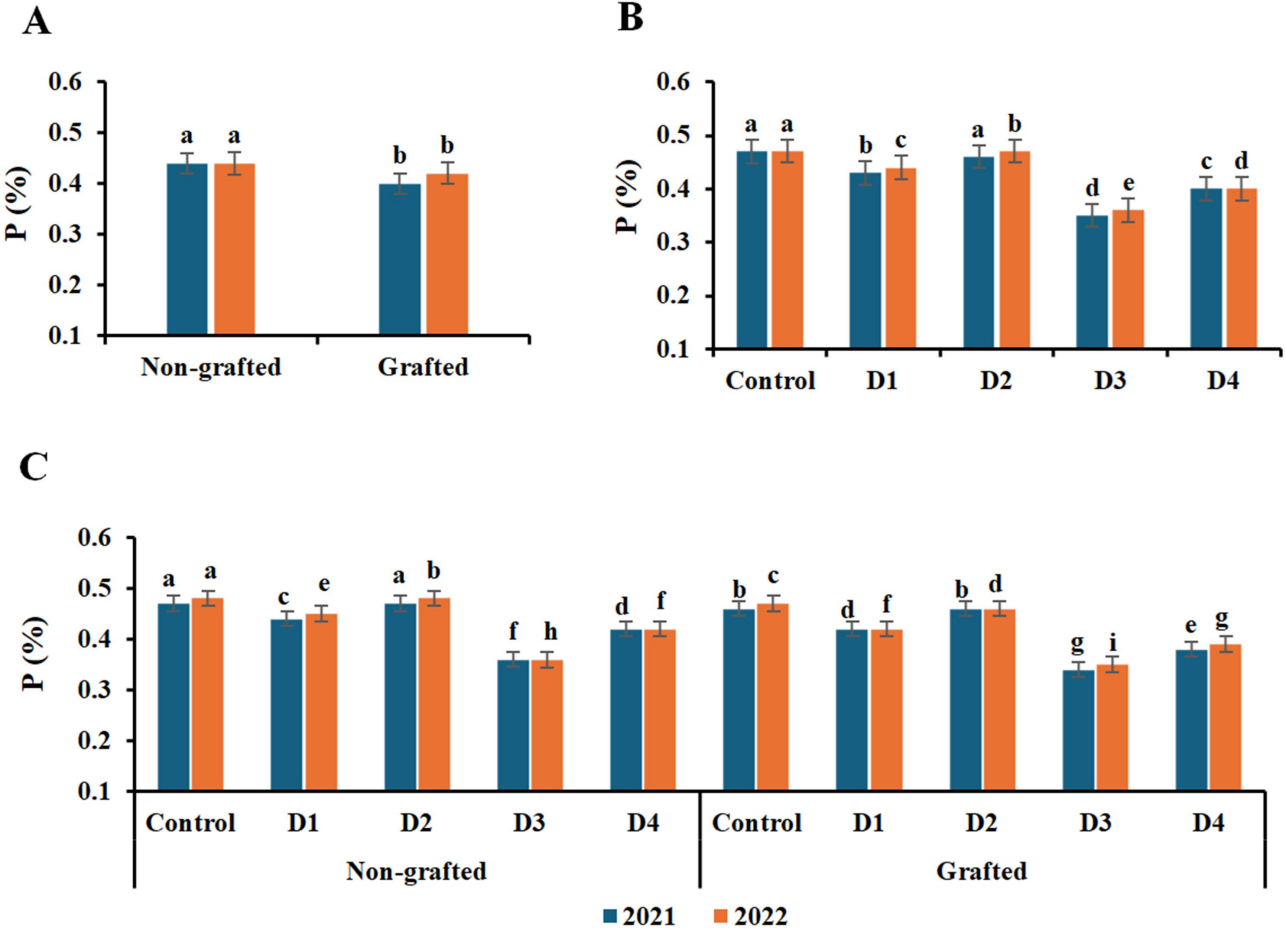
Effect of the grafting **(A)**, irrigation levels **(B)**, and their combination **(C)** on P content during both seasons. Control; 14000 m^3^ ha^−1^), D1 (75% of control; 10500 m^3^ ha^−1^), D2 (75% of control except berries enlargement stage like control; 12000 m^3^), D3 (50% of control; 7000 m^3^ ha^−1^), D4 (50% of control except berries enlargement stage like control; 10000 m^3^); Different letters indicate significant differences among treatments at test (*P* < 0.05). Data are presented as means of three replicates (n = 3) ± standard error (SE).

**Figure 5:**
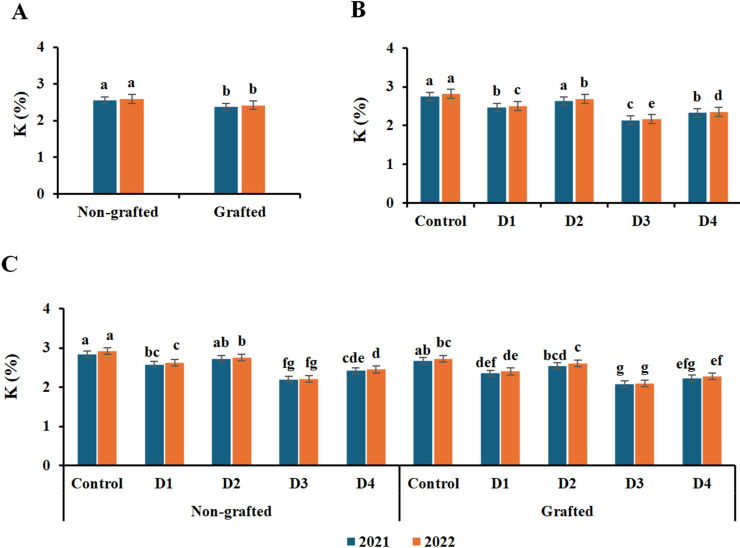
Effect of the grafting **(A)**, irrigation levels **(B)**, and their combination **(C)** on K content during both seasons. Control; 14000 m^3^ ha^−1^), D1 (75% of control; 10500 m^3^ ha^−1^), D2 (75% of control except berries enlargement stage like control; 12000 m^3^), D3 (50% of control; 7000 m^3^ ha^−1^), D4 (50% of control except berries enlargement stage like control; 10000 m^3^); Different letters indicate significant differences among treatments at test (*P* < 0.05). Data are presented as means of three replicates (n = 3) ± standard error (SE).

The leaf contents of N, P, and K macronutrients were significantly affected by grafting, irrigation treatments, and their interaction (**Figures 3**–**5**). The highest contents of N, P, and K were recorded in nongrafted vines in both seasons. The elements followed a similar trend with irrigation treatments, as the control and deficit irrigation D2 recorded the highest nutrient contents in both seasons, whereas D3 showed the lowest contents in both seasons. Similarly, the control and D2 treatments, together with nongrafted vines, recorded the highest N, P, and K contents in both seasons. In contrast, the lowest were recorded under deficit irrigation D3 in both nongrafted and grafted vines.

### 3.4 Physical characteristics of fruits

**Table 3** demonstrates that grafting, irrigation treatments, and their interaction had a substantial impact on berry removal force, the weight of 50 berries, firmness, and diameter. The grafted vines showed the highest fruit firmness in the first season, with no discernible changes in the second season. On the other hand, nongrafted vines showed the highest values of berry removal force, the weight of 50 berries, and diameter in both seasons. In both seasons, the irrigation treatments had no discernible impact on removal force, except D3 in the first season, which produced a noticeably lower figure. The combination of D4 irrigation treatment with nongrafted vines recorded the highest removal force in terms of interaction, with no discernible variations from the D1 and control treatments on nongrafted vines and from D1 on grafted ones. On the other hand, D2 on grafted vines showed the lowest value. Additionally, in both seasons, the control treatment recorded the highest fruit firmness, with no discernible differences from the D1 and D2 irrigation treatments in the second season. Regarding the interaction, the combination of grafted vines with the control treatment in the first season produced the greatest firmness value; however, in the second season, there were no significant differences among all interactions except D4 with grafted vines, which produced the lowest fruit firmness. Furthermore, in both seasons, the control treatments recorded the highest weight of 50 berries, whereas D3 recorded the lowest. Similarly, the interaction between nongrafted vines and the control irrigation treatment showed the highest weight of 50 berries in both seasons; however, in the first season, there were no significant differences compared with D2 and D4 on nongrafted vines and the control on grafted vines. Furthermore, the control treatment had the largest berry diameter in both seasons; however, no discernible changes were found among the other treatments, except for D3, which had the smallest fruit diameter in both seasons. Similarly, in both seasons, the control treatment with nongrafted vines had the largest berry diameter; however, there were no significant differences compared with the combination of nongrafted vines with D1 and D2 in both seasons and D3 in the second season. On the other hand, in both seasons, D3 on grafted vines had the smallest berry diameter.

**Table 3:** Effect of grafting, irrigation levels, and their combination on the weight of 50 berries, berry diameter, firmness, and removal force during both seasons.

Treatment	Weight of 50 berries (g)		Berry diameter (mm)		Firmness (N/cm^2^)		Removal force (N)	
	2021	2022	2021	2022	2021	2022	2021	2022
Nongrafted	212.06 a	197.8 a	17.25 a	17.30 a	397.16 b	412.33 a	653.5 a	642.66 a
Grafted	187.1 b	180.23 b	15.84 b	16.55 b	443 a	396 a	601 b	581.33 b
*F*-test	^**^	^*^	^**^	^*^	^**^	ns	^**^	^**^
Control	237.66 a	228.58 a	17.48 a	17.40 a	472.5 a	447.5 a	632.5 ab	628.33 a
D1	200.16 b	185.25 b	16.83 b	16.89 ab	421.66 b	420.83 ab	625.83 ab	630.83 a
D2	191.16 b	176.41 b	16.32 bc	16.85 ab	420.83 b	430.83 ab	652.08 a	585 a
D3	166.25 c	174.75 b	15.77 c	16.47 b	411.66 b	374.16 bc	590 b	584.16 a
D4	202.66 b	180.08 b	16.32 bc	17.1 ab	373.75 b	347.5 c	635.83 a	631.66 a
*F*-test	^**^	^**^	^**^	^*^	^**^	^**^	^*^	ns
Nongrafted								
Control	239.33 a	239.16 a	17.91 a	18.15 a	436.66 b	448.33 a	646.66 ab	656.66 ab
D1	223.5 ab	193 bc	17.61 ab	17.03 abc	411.66 bc	411.66 a	603.33 bc	633.33 abc
D2	210.66 bc	177 cd	17.61 ab	17.05 abc	405 bc	450 a	707.5 a	650 abc
D3	171.33 de	190.5 bcd	16.36 cde	16.78 bc	383.33 bc	376.66 ab	608.33 bc	590 bcd
D4	215.5 abc	189.33 bcd	16.73 cd	17.5 ab	349.16 c	375 ab	701.66 a	683.33 a
Grafted								
Control	236 ab	218 ab	17.05 bc	16.66 bc	508.33 a	446.66 a	618.33 bc	600 bc
D1	176.83 de	177.5 cd	16.05 de	16.75 bc	431.66 b	430 a	648.33 ab	628.33 abc
D2	171.66 de	175.83 cd	15.02 g	16.66 bc	436.66 b	411.66 a	596.66 bc	520 d
D3	161.16 e	159 d	15.17 fg	16.16 c	440 b	371.66 ab	571.66 c	578.33 cd
D4	189.83 cd	170.83 cd	15.90 ef	16.51 bc	398.33 bc	320 b	570 c	580 cd
*F*-test	^**^	^*^	^*^	^*^	^*^	^*^	^**^	^*^

Control, 14,000 m^<s>3</s>^ ha^−1^; D1, 75% of control (10,500 m^3^ ha^−1^); D2, 75% of control except at the berries enlargement stage, similar to control (12,000 m^3^ ha^−1^); D3, 50% of control (7,000 m^3^ ha^−1^); D4, 50% of control except at the berries enlargement stage, similar to control (10,000 m^3^); N, Newton.

Different letters indicate significant differences among treatments at test (*p* < 0.05). Data are presented as means of three replicates (*n* = 3). *Significant at 0.05 level, **Significant at 0.01 level according to F-test.

### 3.5 Effects on the chemical characteristics of fruits

**Figure 6:**
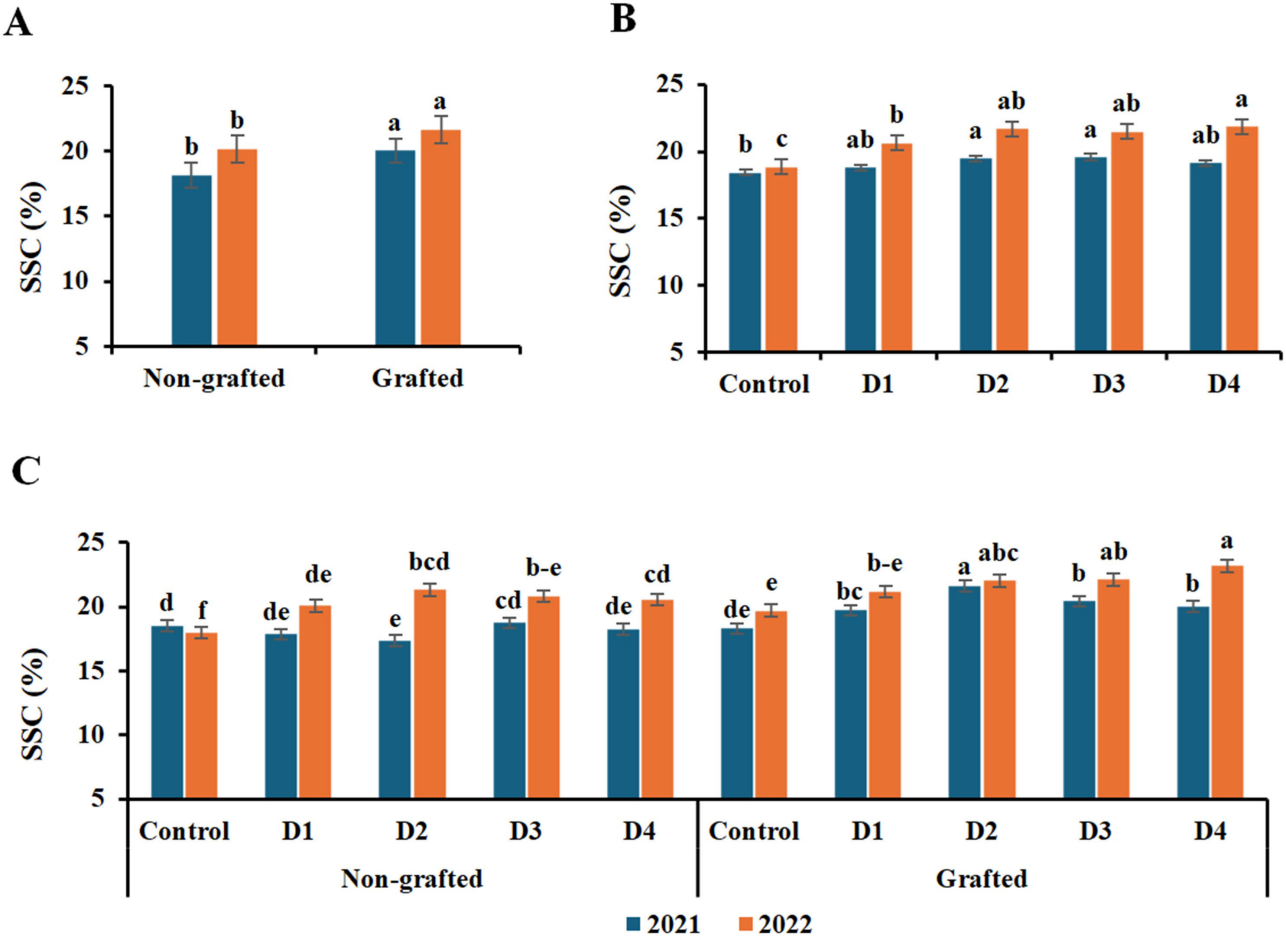
Effect of the grafting **(A)**, irrigation levels **(B)**, and their combination **(C)** on SSC during both seasons. Control; 14000 m^3^ ha^−1^), D1 (75% of control; 10500 m^3^ ha^−1^), D2 (75% of control except berries enlargement stage like control; 12000 m^3^), D3 (50% of control; 7000 m^3^ ha^−1^), D4 (50% of control except berries enlargement stage like control; 10000 m^3^); Different letters indicate significant differences among treatments at test (*P* < 0.05). Data are presented as means of three replicates (n = 3) ± standard error (SE).

**Figure 7:**
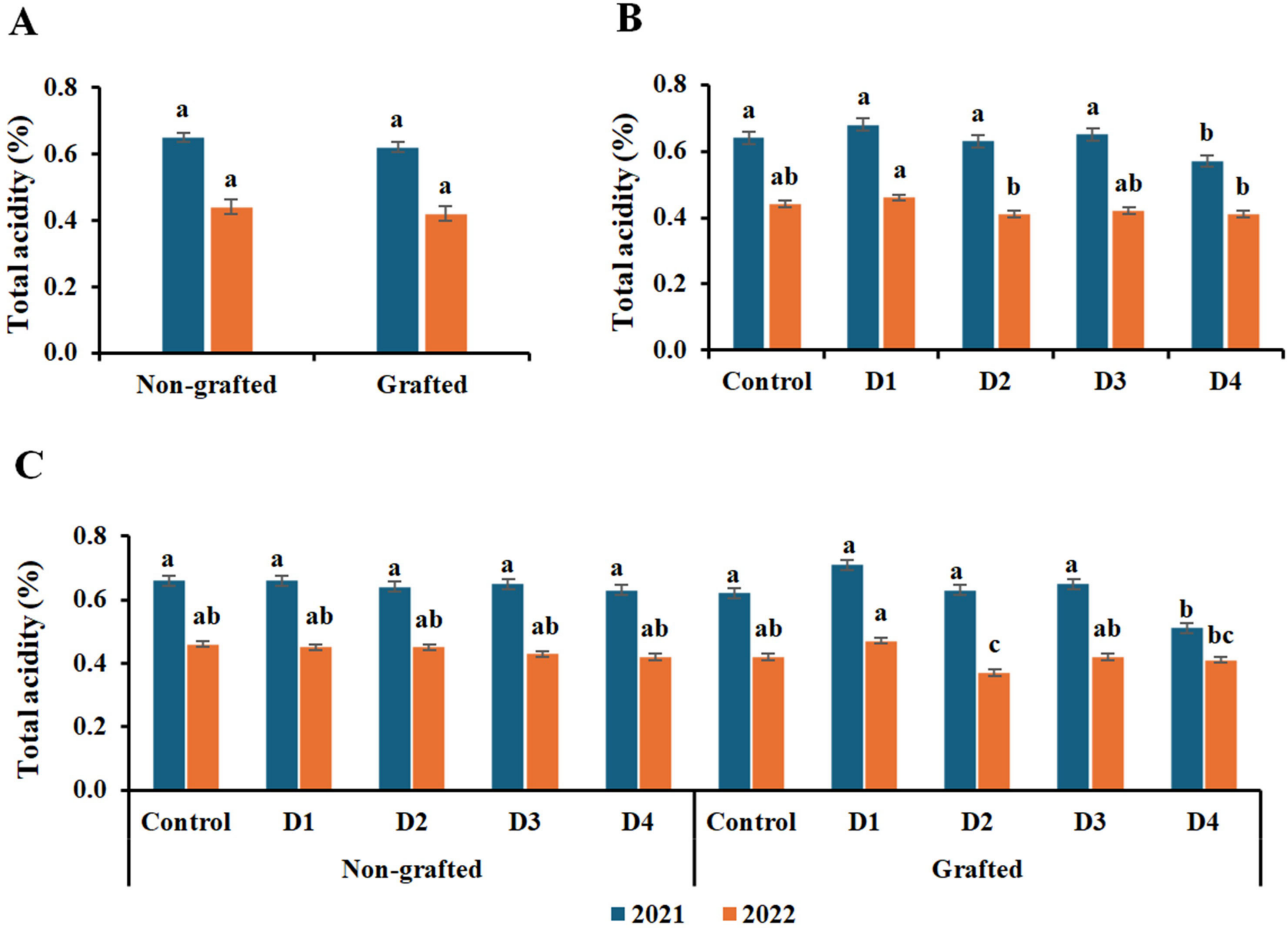
Effect of the grafting **(A)**, irrigation levels **(B)**, and their combination **(C)** on total acidity during both seasons. Control; 14000 m^3^ ha^−1^), D1 (75% of control; 10500 m^3^ ha^−1^), D2 (75% of control except berries enlargement stage like control; 12000 m^3^), D3 (50% of control; 7000 m^3^ ha^−1^), D4 (50% of control except berries enlargement stage like control; 10000 m^3^); Different letters indicate significant differences among treatments at test (*P* < 0.05). Data are presented as means of three replicates (n = 3) ± standard error (SE).

**Figure 8:**
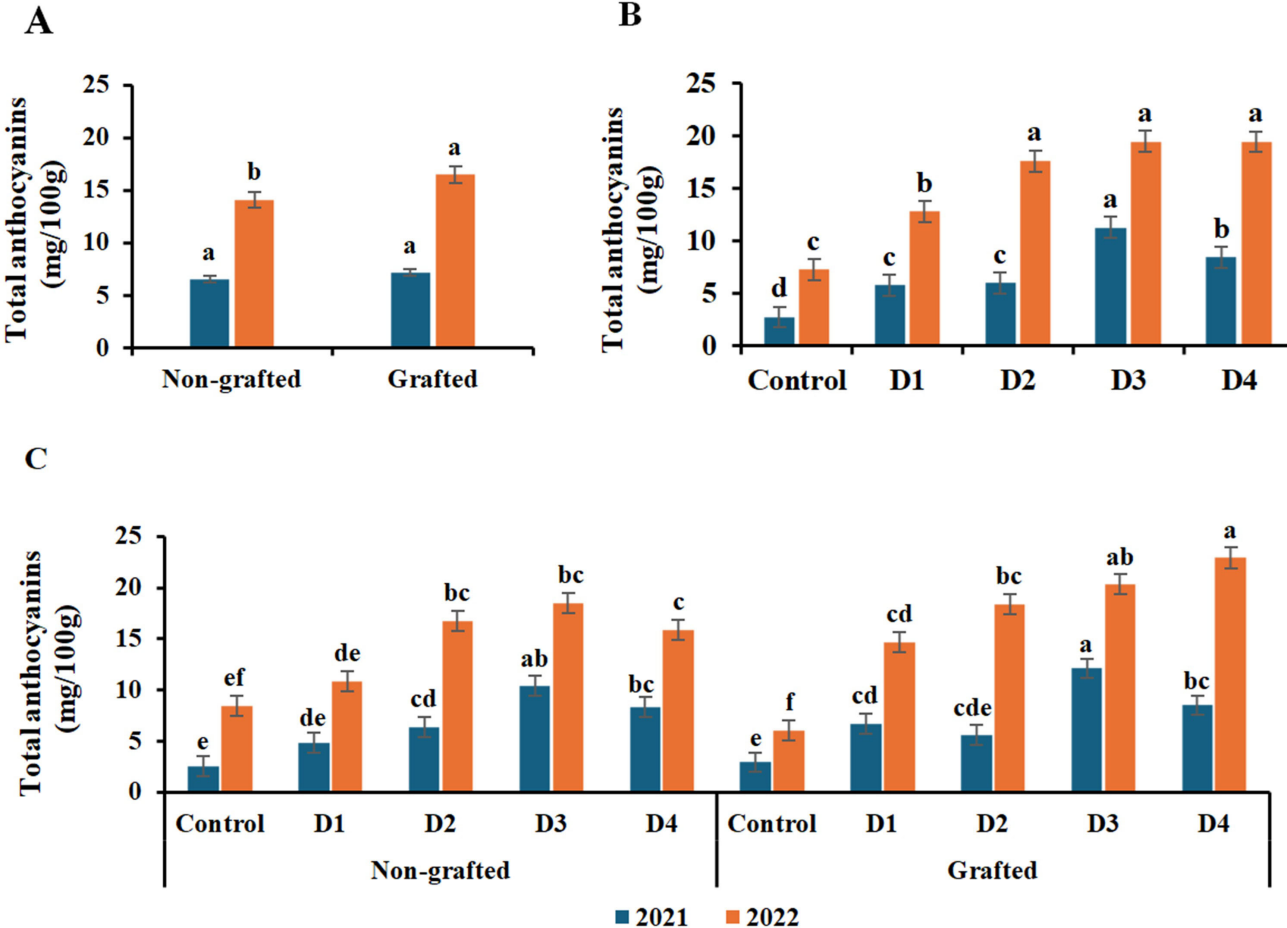
Effect of the grafting **(A)**, irrigation levels **(B)**, and their combination **(C)** on total anthocyanins during both seasons. Control; 14000 m^3^ ha^−1^), D1 (75% of control; 10500 m^3^ ha^−1^), D2 (75% of control except berries enlargement stage like control; 12000 m^3^), D3 (50% of control; 7000 m^3^ ha^−1^), D4 (50% of control except berries enlargement stage like control; 10000 m^3^); Different letters indicate significant differences among treatments at test (*P* < 0.05). Data are presented as means of three replicates (n = 3) ± standard error (SE).

The data in **Figures 6**–**8** demonstrate that grafting, irrigation, and their combination in both seasons significantly impacted SSC, TA, and anthocyanin content in ‘Crimson Seedless’ grapevines. The highest levels of SSC and anthocyanins were observed in grafted grapevines in both seasons; however, the differences in anthocyanin levels were not significant in the first season. In both seasons, no significant difference in TA was observed between grafted and nongrafted vines. With respect to the treatments, SSC increased as deficit irrigation levels increased. D3 had the highest SSC value in the first season and D4 in the second, whereas the control had the lowest values in both seasons. Furthermore, the interaction of grafted vines with D2 in the first season and with D4 in the second season had the highest significant SSC; however, there were no appreciable differences between the combination of grafted vines with D2, D3, and T4 in the second season. Moreover, there were no appreciable differences in TA among irrigation treatments, except D4 in the first season and D2 and D4 in the second season, which had the lowest TA value. Likewise, D4 with grafted vines in the first season was the only interaction that showed a significantly lower TA. However, in the second season, D1 had the greatest TA value, with no discernible variations except for D2 and D4 with grafted vines, which had the lowest TA value. Regarding the effect of irrigation treatments on anthocyanins, it is similar to SSC, as anthocyanin levels rose as the water shortage increased. D3 exhibited the highest significant anthocyanins for both seasons; however, there was no significant difference between D4 and D2 in the second season, while the lowest anthocyanin content was recorded by the control treatment. In terms of interaction, the combination of grafted grape vines with D3 in the first season and with D4 in the second season had the highest significant levels of anthocyanins. On the other hand, the lowest value of anthocyanins was recorded by the interaction of nongrafted and grafted vines with the control in both seasons.

### 3.6 Yield components

The data in **Table 4** illustrate the fruit yield, crop water productivity, income, and irrigation cost as the average of two seasons. The nongrafted vines produced the highest significant total yield compared to the grafted ones. Moreover, D2 treatment produced the maximum yield, without a significant difference from the control, and the lowest was recorded by D3. Regarding the interaction, D2 with nongrafted vines produced the significantly highest total yield compared to other interactions, except the combination of the control with the nongrafted vines, while the lowest was produced by grafted vines with D4. In addition, nongrafted vines gave a significantly higher income per tree compared to grafted ones. Furthermore. D2 recorded the highest significant income per tree, while D4 revealed the lowest. Regarding crop water productivity, D3 gave the highest significant value, followed by D1, D2, and D4 treatments, while the control recorded the lowest. Deficit irrigation treatments saved 25%, 13%, 50%, and 26% of the irrigation water and subsequently reduced fuel costs in the same order for D1, D2, D3, and D4, respectively, compared to the control treatment.

**Table 4:** Fruit yield, income, crop water productivity, and irrigation cost (average of two seasons).

Treatment	Total yield (kg/tree)	Income (EGP/tree)	Crop water productivity (kg/m^3^)	Irrig. cost (EGP/ha)
Nongrafted	17.6 a	529.6 a	3.0 a	
Grafted	16.9 b	507.1 b	3.1 a	
*F*-test	^*^	^***^	ns	
Control	18.3 a	551.4 b	2.3 c	12,978 a
D1	16.8 b	504.9 d	3.1 b	9,729 c
D2	18.8 a	564.7 a	2.8 b	11,278 b
D3	16.9 b	509.9 c	4.3a	6,530 e
D4	15.3 c	460.8 e	2.8 b	9,663 d
*F*-test	^***^	^***^	^***^	^***^
Nongrafted				
Control	19.3 ab	579.8 b	2.3 e	
D1	15.9 d	478.4 h	2.6 cde	
D2	20.5 a	616 a	3.0 bcd	
D3	15.6 d	469.5 i	3.7 b	
D4	16.8 cd	504.2 g	2.2 bc	
Grafted				
Control	17.4 bcd	523.0 e	2.2 e	
D1	17.7 bcd	531.4 d	3.4 b	
D2	17.1 cd	513.5 f	2.5 cde	
D3	18.3 bc	550.3 c	4.9 a	
D4	13.9 e	417.4 j	2.4 de	
*F*-test	^***^	^***^	^**^	

Control, 14,000 m^<s>3</s>^ ha^−1^; D1, 75% of control; 10,500 m^3^ ha^−1^; D2, 75% of control except berries at the enlargement stage, similar to control; 12,000 m^3^ ha^−1^); D3, 50% of control (7,000 m^3^ ha^−1^); D4, 50% of control except at the berries enlargement stage, similar to control (10,000 m^3^ ha^−1^).

Different letters indicate significant differences among treatments at test (*p* < 0.05). *Significant at 0.05 level, **Significant at 0.01 level according to F-test.

Income was calculated by multiplying total yield by the grape price in EGP (based on the farm-gate price of 30 EGP per kg). Crop water productivity was calculated as explained in the Materials and methods. Irrigation cost was calculated based on the diesel fuel price used for irrigation.

## 4 Discussion

The present study was designed to determine the effect of DI and grafting on the production and fruit quality of ‘Crimson Seedless’ grapevines, as well as on some soil attributes under semiarid conditions.

### 4.1 Deficit irrigation and soil properties

Water deficit can alter all soil pathways, including the physical, chemical, and biological attributes, leading to reduced soil quality, impaired ecosystem functioning, and diminished grapevine productivity. Concerning changes in soil physical properties, water deficit might alter the structural and hydrological behavior of soil by reducing soil porosity and hydraulic conductivity, increasing bulk density, and limiting water flow. The macroaggregate proportion and soil aggregate stability could decline under drought (Zhang et al., 2023). Water deficit can also drive distinct changes in soil chemical attributes, including reducing soil organic carbon, total NPK under water deficit (Bghbani-Arani and Poureisa, 2024). Regarding soil biological changes under water deficit, this stress can profoundly affect soil microbial life by reducing microbial biomass, activity, and diversity in many soils, along with decreasing enzyme activities and slowing nutrient and carbon cycling (Hopkins et al., 2024). In the current study, the most obvious finding to emerge from the analysis is that a significant decrease in SOM, CaCO_3_ content, and CEC can be observed due to a decrease in soil moisture content, which restricts many chemical reactions in soil. However, the observed difference between soil total porosity and hydraulic conductivity under DI stress was not significant. Therefore, optimizing irrigation should be carefully managed to avoid excessive water stress, especially under deficit irrigation and soils with high bulk density. Furthermore, monitoring soil physical attributes (mainly regular assessment of bulk density) can help to adjust irrigation strategies to maintain soil health and crop productivity. Our study focuses on the impacts of water deficit on soil at different levels (100%, 75%, and 50%), where the first level (100%) supports stable soil physical and biochemical properties, preventing drought−induced stress; the level of 75% reduces water use while largely sustaining soil chemical, biological, and physical properties; while the level of 50% (as severe deficit irrigation) induces moisture stress that weakens soil structure, microbial diversity, nutrient availability, and carbon cycling. Similar findings were also reported by Zhong et al. (2025).

It is reported that lower values of soil bulk density can promote and increase most of the tomato quality variables under deficit irrigation (Lu et al., 2021). The reason may be soil compaction, which leads to a higher bulk density and limits the growth of tomato roots, thus affecting whole-plant growth. Concerning SOM, soil organic matter management practices can improve water use efficiency and soil health. DI can influence the formation of the new SOM. SOM plays a crucial role in mitigating the negative effects of water stress associated with DI. A possible explanation for this might be that SOM controls water retention and soil structure (Núñez et al., 2022). Increasing DI can decrease SOM due to the reduced soil moisture. Altered soil moisture-sensitive biochemical pathways can cause shifts in soil enzyme activities, microbial metabolism, and decomposition dynamics that accelerate SOM loss. The mechanisms are biochemical in nature, centered on carbon metabolism, enzyme-mediated reactions, and microbial resource allocation. This leads to more respired carbon as CO_2_ and less being incorporated as microbial biomass, as major components of stable SOM (Hai et al., 2022). Under DI, rhizosphere bacteria such as Actinobacteria and Proteobacteria, which include strong decomposers, became more abundant, and communities then shifted to fast−cycling, enzyme-rich decomposers that accelerate SOM mineralization (Qin et al., 2025). Medium irrigation (drier conditions than high irrigation) increased C-acquiring enzyme activities, stimulating SOM decomposition (Ye et al., 2022), whereas serious deficit irrigation might increase urease activity, signaling intensification of biochemical N and C turnover (Ning et al., 2024). These results are consistent with those of other studies and suggest that DI, along with organic mulching, enhanced mango productivity and yield under arid climatic conditions (Alhashimi et al., 2023a).

### 4.2 Response of grapevines to deficit irrigation and grafting

In the present study, chlorophyll (SPAD value), leaf area, and dry weight increased or remained unaffected under the stressful treatments. Similar results were confirmed by Salama et al. (2024) on mandarin trees, where dry weight was not affected by water deficit treatments. Grapevine is classified as relatively tolerant to drought (Zakalashvili et al., 2024), which may explain why some vegetative growth parameters are unaffected under deficit irrigation. On the other hand, Alhashimi et al. (2023b) reported that a negative correlation between SPAD values and leaf water content might explain why dry weight and chlorophyll content increased or remained unaffected under deficit irrigation. However, the differences in dry weight in the present study were not significantly affected. The deficit irrigation treatments in the present investigation reduced the plant contents of NPK. The reduction of leaf NPK content in deficit irrigation treatments may be linked to the availability of moisture, which aids in nutrient uptake (Amare et al., 2024). They reported that the uptake of NPK increased with increasing irrigation level, but excessive irrigation might be useless or may have negative effects on plant NPK contents.

Fruit weight, diameter, and firmness decreased due to deficit irrigation treatment in the present study. These findings are consistent with those reported by Faghih et al. (2021), who found that apple fruit weight dropped when water restriction was applied. This might be due to the high amounts of ABA generated in such circumstances, which may affect fruit weight and, in turn, fruit diameter and yield (Kobashi et al., 1997; Alhashimi et al., 2023c; Salama et al., 2024). Furthermore, fruit firmness decreased with deficit irrigation treatments in the present study. Similar results were reported in apple (Faghih et al., 2021); fruit firmness increased more in the control than in moderate water-stress treatments. The water relations of trees could affect the turgor of fruit cells and their chemical and mechanical properties (Salama et al., 2023). The decrease in fruit weight may be the cause of the yield drop brought on by some water shortage treatments, especially under severe deficit irrigation (Faghih et al., 2021). However, the total yield was not significantly affected by deficit irrigation D2 and D1 in the first season and by all deficit irrigation treatments in the second season, with water savings.

In the present study, deficit irrigation significantly enhanced the grape SSC. SSC is a key indicator of grape quality, as it is typically used by exporters and importers worldwide. Moreover, it is influenced by acidity, which affects the flavor of grape berries. According to similar findings, water stress raised SSC levels and decreased fruit acidity in apples (Wang et al., 2019). Similarly, Ezzat et al. (2021) and Salama et al. (2024) reported that the fruit SSC rose as a result of deficit irrigation in apricot and mandarin trees, respectively. Deficit irrigation might inhibit the enzymes pectinase and polygalacturonase, whereas water stress could promote the production of ethylene, leading to the accumulation of glucose, fructose, and sucrose (Ebel et al., 2019; Silva et al., 2021).

Anthocyanins are responsible for the red color and, subsequently, the appearance quality of ‘Crimson Seedless’ grape (Salama et al., 2023), as well as being valuable ingredients of functional foods (Stanca et al., 2024). The anthocyanin contents were greatly enhanced by deficit irrigation treatments, as total anthocyanin contents increased with increasing water scarcity. Previous studies reported the effect of water deficiency stress on anthocyanin accumulation (Geng et al., 2025; Tan et al., 2025a). The increase in anthocyanins under deficit irrigation could be due to an increase in some monomeric anthocyanins, including delphinidin-3-*O*-glucoside and (Del-3-O) cyanidin-3-O-glucoside, which were reported by Tan et al. (2025a). Deficient irrigation could promote the synthesis and related gene expression of anthocyanins (Geng et al., 2025). The more anthocyanins there are, the deeper the red color, which greatly improves fruit appearance and subsequently results in a higher price.

Grafted grapevines produced the maximum values of leaf chlorophyll contents, fruit firmness, SSC, and anthocyanin contents, while the nongrafted vines produced the highest leaf area, leaf dry weight, NPK content, fruit weight and diameter, and removal force. Fruit yield and titratable acidity did not reveal significant differences between grafted and nongrafted vines. Varieties differ regarding the effect of certain rootstocks on vegetative growth, fruit yield, and quality. Klimek et al. (2022) reported that fruit parameters and yield were not significantly affected by grafting on SO_4_ rootstock compared with nongrafted vines, except for SSC, which increased in grafted grapevines. The benefits of rootstocks or grafting depend on soil conditions, region, and cultivar. Rootstocks could be an approach for mitigating drought stress in grapevines (Verslype et al., 2023), which might contribute to saving water in semiarid regions. The interaction between certain rootstocks and scions depends on the cultivar, soil type, properties, and environmental factors. In the present study, 110 Richter rootstocks revealed dwarfing effects on ‘Crimson seedless’ cultivar, where they produced lower vegetative growth parameters but improved most of the fruit quality parameters, without significant effects on fruit yield. However, rootstocks could mitigate the negative effects of drought stress and increase plant adaptability to environmental factors.

### 4.3 Yield components

The results of this research revealed a positive impact of deficit irrigation on crop water productivity and lower irrigation costs. On the other hand, the average yield was not affected by the light deficit irrigation (D2). Water is a very vital and limited natural resource, especially in semiarid regions like Egypt. Saving water means increasing the cultivated area and production by the same percentage (saving 25% water increases area and production by 25%) with a fixed amount of water. However, the benefits are not only saving water but also minimizing the negative impacts of using diesel on the environment, as well as enhancing water use efficiency. Moreover, the deficit irrigation treatments significantly reduced irrigation costs and subsequently increased net income for growers. Similar results were reported on the impacts of deficit irrigation on pomegranates (Hassan et al., 2025), where it was reported that the more deficit irrigation was applied, the greater crop water productivity and net income for each tree. Recent studies revealed some applications that could mitigate drought stress and improve plant vegetative growth, fruit yield, quality, and crop water productivity under deficit irrigation stress (Abdel-Sattar et al., 2024; El-Sayed et al., 2024; Zapata-García et al., 2024; Salama et al., 2026). The application of proline to Hundz soil improved vegetative and fruit quality attributes of ‘Crimson Seedless’ under deficit irrigation conditions (El-Sayed et al., 2024). Also, Zapata-García et al. (2024) reported that the application of biostimulants under deficit irrigation treatments enhanced mycorrhization rate and, subsequently, grape root growth, resulting in improved water productivity and precocity of yield. Moreover, the application of 24-epibrassinosteroids (Br) and hydrogen peroxide improved the ‘Keitt’ mango trees’ leaf mineral and chlorophyll content, fruit set, yield, and quality under deficit irrigation stress (Abdel-Sattar et al., 2024). Research on these types of applications and technologies could help advance the deficit irrigation applications without negative effects on plant vegetative growth, fruit yield, and quality.

## 5 Conclusions

Grapevine production in arid regions faces the significant challenge of water scarcity, making it necessary to find an efficient irrigation method. The present findings indicate that deficit irrigation influences selected soil attributes and vine performance. Among physical and chemical soil properties, soil bulk density and SOM are more sensitive to DI. While soil bulk density does not directly cause DI, it influences how soils respond to reduced water applications. Higher soil bulk density can worsen the effects of DI by reducing water availability to plants, whereas proper soil management can mitigate these challenges. Moderate and light deficit irrigation did not significantly affect vegetative growth or yield while improving fruit quality traits, particularly soluble solids content and anthocyanin accumulation, in addition to enhancing water productivity. Reduced irrigation inputs also lowered irrigation costs and improved overall water use efficiency. Overall, the results suggest that light and moderate deficit irrigation can be a practical strategy in vineyards under semiarid conditions, especially when combined with appropriate rootstock to mitigate the negative effects of drought. Nevertheless, further research is recommended to explore additional sustainable technologies and management practices that may enhance the effectiveness of deficit irrigation strategies under increasing water scarcity.

## Data Availability

The original contributions presented in the study are included in the article/supplementary material. Further inquiries can be directed to the corresponding author.
